# Methanotrophic microbiomes for dilute coal-mine methane biofiltration: ecophysiological constraints and design priorities

**DOI:** 10.3389/fmicb.2026.1953995

**Published:** 2026-09-11

**Authors:** Junjie Wei, Haoyang Ren

**Affiliations:** School of Intelligent Engineering, Shandong Management University, Jinan, China

**Keywords:** biofilm mass transfer, biofiltration, coal-mine methane, low-concentration methane, methanotrophic microbiome, microbial consortia, ventilation air methane

## Abstract

Dilute methane from coal-mine ventilation, drainage systems, and abandoned workings is difficult to treat because low methane partial pressure coincides with high or variable gas flow, short feasible residence times, fluctuating temperature and humidity, nutrient scarcity, and dust or trace contaminants. Aerobic methanotrophs can oxidize methane under moderate conditions, but mine-relevant performance cannot be inferred from isolate activity or gas-phase removal efficiency alone. This Mini Review organizes evidence by coal-mine source class and examines how methanotroph ecophysiology, partner organisms, carrier properties, hydrated biofilm structure, and gas transfer constrain sub-percent methane treatment. Mine-targeted studies comprise laboratory gas-phase reactors, mine-derived enrichments, batch experiments, and recent devices; no long-term validation under authentic mine-gas operation was identified in the targeted literature search. Transferable evidence comes from packed-bed reactors, landfill and compost systems, soils, and defined communities. These studies identify candidate traits that require mine-specific testing, including activity at the target methane partial pressure, recovery after starvation, nutrient economy, attachment, and stress tolerance. We propose source-matched functional microbiomes, activity-resolved methane balances, structured carriers, standardized reactor metrics, and explicit, site-specific no-go conditions as development priorities. Biofiltration should not advance where safe oxygen availability, practicable residence time, acceptable backpressure, or sustainable water and nutrient supply cannot be reconciled with mine operation.

## 1 Introduction

Coal mining releases methane through ventilation air, gas drainage, exposed coal surfaces, and abandoned workings. Here, ventilation air methane (VAM) denotes the oxygen-rich, highly dilute exhaust of mine ventilation; drainage gas denotes methane recovered by degasification systems, ranging from methane-rich, oxygen-poor streams to diluted mixtures; and abandoned-mine emissions denote releases from closed workings through vents, shafts, fractures, or diffuse pathways. This Mini Review considers aerobic methane oxidation only where gas composition and operating conditions permit safe oxygen access. A functional methanotrophic microbiome is defined as a carrier-associated community in which methane oxidation is directly demonstrated and any proposed partner function is supported by activity-resolved measurements, perturbation, metabolite evidence, or reproducible consortium reconstruction. Gas-phase CH_4_ disappearance is not equated with biological oxidation unless supported by methane mass balance or complementary activity evidence; community composition or co-occurrence alone is insufficient. Anaerobic methane oxidation is outside the scope of oxygen-rich gas-phase biofilters.

Early gas-phase studies targeted coal-mine applications, but their feed concentrations and residence times were not always representative of VAM (Apel et al., 1991; Sly et al., 1993). Coal-packed biofilters later demonstrated attached-community activity at 1% CH_4_ (Limbri et al., 2014), while mine-derived enrichments broadened the inocula available for testing (Jiang et al., 2016). Mine-scale deployment has not followed. Reactor performance emerges from methane and oxygen transfer, moisture, nutrients, biofilm development, disturbance, and the operating envelope rather than from inoculum or packing alone.

**FIGURE 1:**
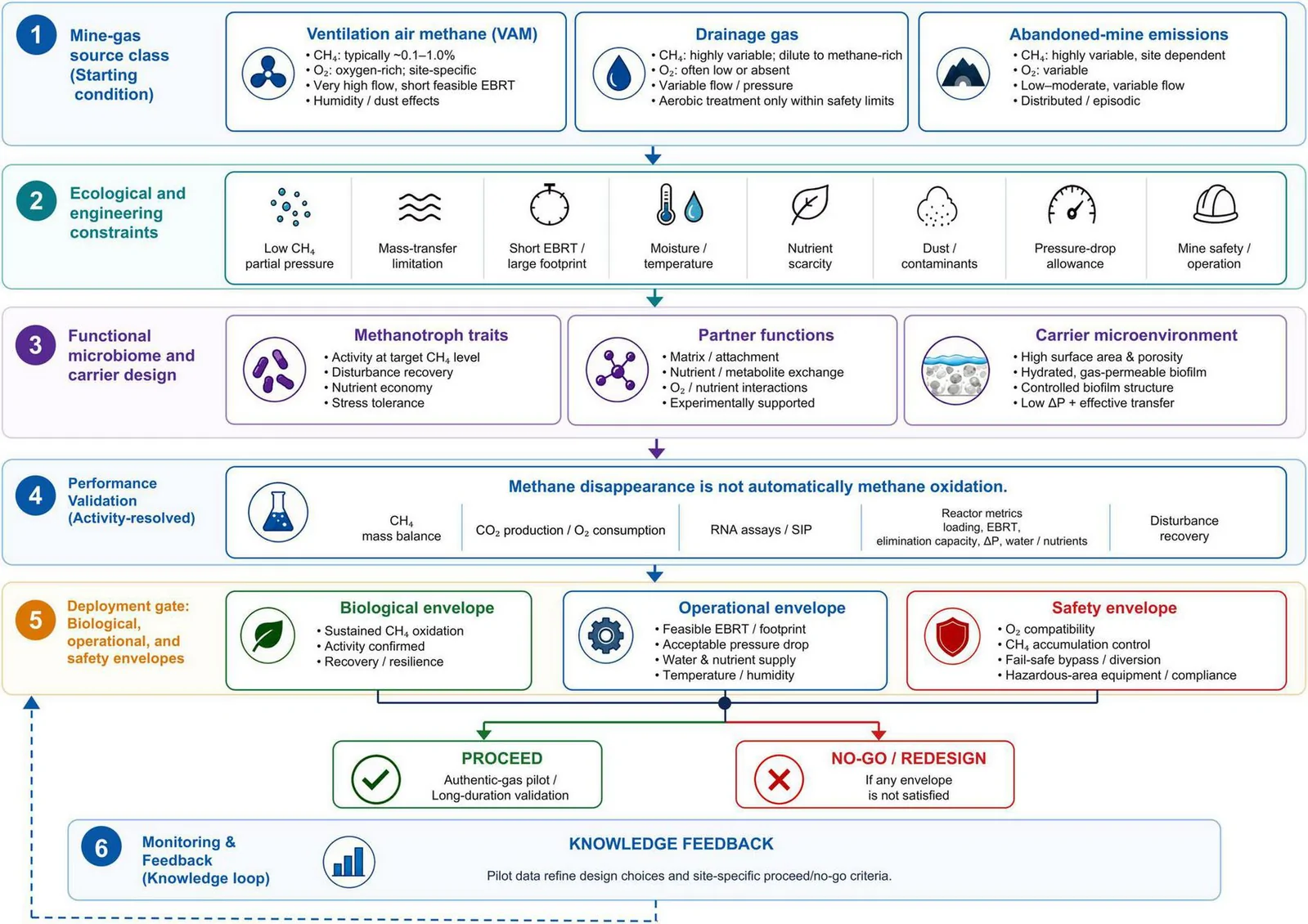
Source-matched decision framework for dilute coal-mine methane biofiltration. Coal-mine source class first defines CH_4_/O_2_ composition, flow, variability, and gas-train context. Within a candidate treatment window, community selection, carrier structure, moisture and nutrients, and the EBRT–flow trade-off shape a hydrated, permeable, functionally verified carrier-associated microbiome. Performance evaluation separates biological evidence–methane mass balance or activity attribution, elimination capacity, and disturbance recovery–from the operational and safety envelope–EBRT and footprint, pressure-drop allowance, resource logistics, oxygen compatibility, methane-accumulation control, fail-safe bypass, and equipment certification. Candidates satisfying all gates proceed to long-duration authentic-gas pilot validation; candidates exceeding site-specific limits are redesigned or designated no-go. Dashed arrows indicate monitoring-informed feedback.

Earlier reviews focused on VAM-specific biofiltration challenges (Limbri et al., 2013), broader biological and engineering controls on methane biofiltration (La et al., 2018; Ahmadi et al., 2024), removal mechanisms and enhancement strategies (Wu et al., 2025), or reactor designs and low-concentration performance (Moss et al., 2026). The present review uses mine-gas source class as the primary organizing variable. Source composition, oxygen status, flow, variability, and gas-train position first define whether an aerobic treatment window exists; microbial functions and carrier microenvironments are then evaluated within that window, followed by validation metrics and site-specific operational and safety gates. Figure 1 summarizes this source-trait-microenvironment-validation framework.

A targeted narrative search of PubMed and Google Scholar, supplemented by backward citation checking and searches of publisher websites, was updated through July 2026 using combinations of terms for coal-mine methane, ventilation air methane, methanotrophs, methane biofiltration, microbial consortia, biofilms, and carrier materials. Studies were prioritized when they addressed mine relevance, low-concentration operation, continuous-flow testing, quantitative reactor metrics, or activity-resolved microbial measurements. Evidence was interpreted in three tiers: mine-targeted reactors or enrichments, transferable non-mine reactors, and mechanistic studies that inform candidate traits without constituting mine validation. Table 1 summarizes representative studies and their principal translational limitations. Table 2 positions this source-first approach relative to those syntheses. No long-term field or pilot validation under authentic mine-gas operation was identified in this targeted search; because the search was not systematic, this finding should not be read as an exhaustive census.

**TABLE 1:** Representative evidence relevant to dilute coal-mine methane biofiltration.

Evidence class	Methane source and concentration	System and microbial basis	Key operating conditions	Study-reported performance	Main translational limitation	Reference
Mine-targeted laboratory reactor	Laboratory coal-mine ventilation atmosphere; 0.25%–1.0% v/v CH_4_	Continuous gas-phase biofilter; pure culture of *Methylomonas fodinarum*	Gas residence time 5–20 min	Approximately 20% removal at 5 min, >70% at 15 min, and approximately 90% at 20 min	Residence times exceed those feasible for many full-scale ventilation streams	Sly et al., 1993
Mine-targeted laboratory reactor	Simulated ventilation air; 1% v/v CH_4_	Coal-packed biofilter; introduced *Methylosinus* isolate; 16S succession showed abundant *Methylocystis*-related sequences	30 °C; 1% v/v CH_4_; inlet gas flow 1.6 L min^–1^; EBRT 2.4 min	Maximum elimination capacity 27.2 ± 0.66 g CH_4_ m^–3^ h^–1^, corresponding to 19.7% ± 2.9% removal at 1.6 L min^–1^	Laboratory gas and short duration; succession does not identify active oxidizers	Limbri et al., 2014
Mine-derived enrichment	Chinese coal-mine soil; artificial coal-bed gas or methane used for enrichment	Mine-derived liquid enrichment; 16S succession; methanotrophic *Methylomonas* and *Methylocystis* became dominant, while *Methylophilus* was the prevailing methylotroph	Sequential laboratory enrichment under methane-containing gas	Methane-oxidation capacity increased during enrichment in parallel with increased methanotroph abundance	No attached-growth reactor, gas-transfer assessment, or sub-percent continuous-flow validation	Jiang et al., 2016
Mine-targeted laboratory device	Simulated coal-mine gas; 1%–10% v/v CH_4_	Charcoal-packed apparatus; mixed flora; microbial identification and activity attribution were limited	Charcoal particles 0.5–1 mm; 27 °C–34 °C	Maximum value of the study-defined metric “unit methane absorption”: 14.213 μmol cm^–3^ h^–1^	Most concentrations exceed typical ventilation-air methane; the reported metric is not standard elimination capacity	Xie et al., 2025
Transferable non-mine laboratory reactor	Dilute methane; 1% v/v CH_4_	Fly-ash-ceramsite biofilter; mixed community; *pmoA* DNA abundance	Controlled laboratory operation	Average elimination capacity 4.628 g CH_4_ m^–3^ h^–1^, 33.4% higher than the suspended-cell system	Artificial feed and laboratory scale; *pmoA* abundance does not establish causation	Sun et al., 2020
Transferable very-dilute laboratory reactor	Approximately 1,000 ppmv CH_4_	Autoclaved-aerated-concrete biofilter; mixed biofilm; community profiling without population-resolved activity	127-day operation	Mean removal efficiency approximately 28.7%	Non-coal source; flow, pressure-drop, and mine-integration constraints were not tested	Ganendra et al., 2015
Transferable very-dilute laboratory reactor	Synthetic air; 455–515 ppmv CH_4_	Packed-bed column; defined strain *Methylotuvimicrobium buryatense* 5GB1C; reactor mass balance	Biomass established for 1 day at 2.5% CH_4_ before 500-ppmv operation; multiple flow rates and bead sizes	Up to 90% gas-phase removal at approximately 125 cm^3^ min^–1^; maximum elimination capacity 2.1 g CH_4_ m^–3^ h^–1^ at approximately 500 cm^3^ min^–1^	Defined strain and laboratory feed; 0.3-mm beads clogged; mine-relevant pressure drop, disturbance recovery, and long-term performance were not tested	He et al., 2025

Evidence classes distinguish mine-targeted laboratory reactors, enrichments, and devices from transferable non-mine systems. No long-term field or pilot validation under authentic mine-gas operation identified in the targeted search is represented. Values are reproduced as reported and with the metric definitions used by the original studies; differences in concentration, EBRT, loading, temperature, reactor volume, metric definition, and microbial evidence type preclude direct performance ranking. EBRT, empty-bed residence time; ppmv, parts per million by volume.

**TABLE 2:** Positioning of the present Mini Review relative to prior reviews of methane biofiltration and low-concentration methane treatment.

Review	Primary organizing focus	Mine-source differentiation	Microbial ecology/consortia	Carrier, mass transfer, reactor performance	Safety/techno-economic emphasis	Field validation/decision boundary
Limbri et al., 2013	Challenges to VAM biofiltration	VAM specifically	Methanotroph suitability discussed	Biofilter media, transfer, EBRT, and scale-up constraints	Mine/VAM feasibility discussed	Deployment barriers identified; no explicit proceed/no-go gate
La et al., 2018	Critical review of methane biofiltration and controlling factors	Multiple methane contexts; not organized by mine source	Biological and non-biological controls reviewed	Packing media, operating factors, and performance emphasized	Engineering feasibility considered; mine safety not central	Research gaps identified; no source-specific gate
Ahmadi et al., 2024	Biotic and abiotic factors in methane biofiltration	Diffuse methane sources; not organized by mine source	Methanotrophs, associated microbes, fungi, and abiotic effects	Biofilm, packing, moisture, temperature, and nutrients emphasized	General engineering/economic viability noted	General future needs; no mine-specific decision gate
Wu et al., 2025	Removal mechanisms, co-contaminants, applications, and enhancement strategies	Broad gas-biofiltration applications; not organized by mine source	Mechanisms and microbial responses discussed	Biofilter strategies, mass transfer, and performance enhancement emphasized	Practical application discussed; mine-specific safety not central	Future directions; no mine-source proceed/no-go gate
Moss et al., 2026	Low-concentration reactor designs and performance	Low-concentration sources compared mainly by reactor/contacting strategy	Methanotrophic capabilities considered at reactor level	Strong reactor, transport, and operability emphasis	Energy, safety, and scale-up constraints highlighted	Performance and scale-up limits; no explicit mine-source gate
Present Mini Review	Mine-gas source → functional traits → carrier microenvironment → validation gate	VAM, drainage gas, and abandoned-mine emissions differentiated first	Activity evidence separated from composition/genetic potential; partner-function criteria explicit	Carrier/biofilm and transfer evaluated within the source-specific operating window	Biological feasibility separated from operational/safety envelope; techno-economics treated as downstream screening	Direct vs. transferable evidence separated; authentic-gas validation and site-specific proceed/no-go decision explicit

The comparison identifies the primary organizing focus and whether source classification, microbial-function evidence, carrier and mass-transfer constraints, safety or techno-economic considerations, field validation, and explicit decision boundaries are central to each synthesis. Descriptions indicate emphasis rather than study quality.

## 2 Dilute coal-mine methane as an ecological and engineering niche

Ventilation air, drainage gas, and abandoned-mine emissions present different biological and engineering opportunities. VAM combines sub-percent methane, abundant oxygen, and very large, usually continuous flow; its central constraint is therefore obtaining sufficient methane uptake at an EBRT and pressure drop compatible with mine ventilation. Drainage gas spans methane-rich, oxygen-poor streams to diluted mixtures. Methane-rich drainage gas is generally better suited to recovery or conventional utilization, whereas aerobic biofiltration is relevant only to suitably dilute or residual streams whose oxygen status and gas-train position permit treatment without unsafe conditioning. Abandoned-mine emissions can be spatially distributed and temporally variable; where characterization confirms diffuse flux, safe oxygen access, and manageable flow, passive biocovers or modular beds may warrant testing. Each source should therefore be classified by CH_4_ and O_2_ composition, flow and variability, and position in the gas-handling system before aerobic treatment. Conditioning, dilution, recirculation, or diversion must remain within site-specific ventilation, monitoring, and explosion-protection requirements (United Nations Economic Commission for Europe (UNECE), 2025).

Dilute coal-mine methane imposes chronic carbon limitation interrupted by pulses or feed loss. Low methane partial pressure weakens transfer to hydrated surfaces, while high flow shortens contact time and can dry the bed. Temperature, humidity, and dust alter reaction, evaporation, pore access, and wettability. Nitrogen, phosphorus, copper, iron, and other nutrients cannot be assumed from the gas stream. High bottle activity may therefore fail in a reactor where hydration and gas-to-biofilm transport restrict substrate access.

Performance reporting must reflect these constraints. Removal efficiency is a gas-phase metric and can appear favorable at low loading or long empty-bed residence time (EBRT); biological oxidation requires an adequate methane balance or complementary activity evidence. Elimination capacity is interpretable only with inlet concentration, loading, temperature, water content, pressure drop, and EBRT. Recovery after starvation or load shock matters for intermittent sources. At mine scale, fan power and footprint may be more restrictive than peak laboratory removal. Techno-economic and life-cycle conclusions therefore remain sensitive to assumed productivity, pressure drop, and methane concentration (Landgren et al., 2025; Moss et al., 2026).

## 3 Methanotrophic traits governing removal at low methane concentration

Classic aerobic methanotrophs include Type I and Type X Gammaproteobacteria, notably Methylococcaceae, and Type II Alphaproteobacteria, mainly Methylocystaceae and methanotrophic lineages within Beijerinckiaceae. Type I organisms generally use the ribulose monophosphate pathway, whereas Type II organisms rely mainly on the serine cycle. Type X is a historical phenotype-based grouping of mainly thermotolerant gammaproteobacterial methanotrophs, not a formal taxonomic rank; these organisms use the ribulose monophosphate pathway while retaining selected serine-cycle enzymes. Methanotrophs within Verrucomicrobiota form another lineage, although their relevance to neutral-pH coal-mine biofilters is uncertain (Hanson and Hanson, 1996; Guerrero-Cruz et al., 2021; Ahmadi and Lackner, 2024). Taxonomy becomes useful for design when paired with measured affinity, recovery, nutrient demand, attachment, or temperature response.

Methane activation is catalyzed by particulate methane monooxygenase (pMMO) or soluble methane monooxygenase (sMMO). pMMO is copper-dependent and membrane-bound in most characterized aerobic methanotrophs; sMMO occurs in fewer taxa and contains a diiron catalytic center. Copper responses are strain- and condition-dependent (Semrau et al., 2010; Ross and Rosenzweig, 2017). Rare-earth elements can influence XoxF-type methanol dehydrogenases where encoded and expressed, so trace-element additions should be tested as selective pressures rather than universal nutrient recipes (Keltjens et al., 2014; Semrau et al., 2018).

*pmoA*-targeted DNA assays indicate genetic potential and methanotroph biomass rather than current methane-oxidation rate (Smith and Wrighton, 2019). Cell-normalized *pmoA* transcripts correlated with cell-specific oxidation across Type I and Type II methanotrophs under controlled conditions, supporting RNA measurements paired with mass balances (Tentori et al., 2022). Stable-isotope probing can further link populations to function. Low-concentration growth must be distinguished from atmospheric-methane physiology (He and Lidstrom, 2024; Tveit et al., 2025). Alternative pMMO systems in some *Methylocystis* strains can increase affinity, whereas specialized soil methanotrophs oxidize atmospheric methane (Baani and Liesack, 2008; Schmider et al., 2024). Affinity alone does not guarantee reactor productivity. He et al. (2023) demonstrated growth near 500 ppm and below; packed-bed experiments with *Methylotuvimicrobium buryatense* 5GB1C at 500 ppm reported up to 90% gas-phase removal at low flow and a maximum elimination capacity of 2.1 g CH_4_ m^–3^ h^–1^ (He et al., 2025). The defined strain, laboratory feed, pre-establishment at 2.5% methane, and bench scale do not establish mine-compatible pressure drop, disturbance recovery, or long-term productivity.

Ammonium supports biomass synthesis, but the un-ionized NH_3_ fraction increases with pH and can compete with methane for MMO-mediated oxidation. Hydroxylamine formation and subsequent nitrogen transformations depend on methanotroph detoxification, associated nitrogen-transforming microorganisms, and operating conditions (Bodelier and Laanbroek, 2004). Phosphorus can constrain oxidation, while pH and water content affect activity, community composition, hydration, and gas-filled porosity (Veraart et al., 2015; Yao et al., 2023). Thermotolerant *Methylocaldum* may suit self-heating compost beds (Avila-Nuñez et al., 2026), but this does not establish mine-biofilter performance; cold or variable mine streams require direct low-temperature testing.

## 4 From isolates and enrichments to functional methanotrophic microbiomes

Pure cultures remain essential for resolving kinetics, enzyme regulation, and nutrient requirements. *Methylomonas fodinarum* supported an early ventilation-air biofilter (Sly et al., 1993), while mine-derived enrichments and coal-mine acclimation studies broadened candidate inocula (Jiang et al., 2016; Zhou et al., 2023; Xie et al., 2026). Extrapolation remains limited because strains selected in methane-rich batch culture may lose competitiveness under sub-percent methane, drying, temperature shifts, and uneven nutrient delivery.

Methanotroph-associated communities may exchange one-carbon intermediates, organic acids, vitamins, matrix components, and products of cell turnover, but the magnitude and direction of these interactions are taxon- and condition-dependent. In non-mine experimental systems, heterotroph richness, helper strains, or volatile metabolites altered methane oxidation or methanotroph physiology (Ho et al., 2014; Jeong and Kim, 2019; Brenzinger et al., 2024). These observations identify candidate interaction mechanisms rather than validated partner functions in attached coal-mine biofilters; partners may also compete for oxygen and nutrients or increase diffusion resistance. Consistent with the definition above, a functional microbiome claim requires direct methane-oxidation evidence plus experimental support for any attributed partner function. Evidence for a useful trait should not be treated as evidence that a mine-relevant functional microbiome carrying that trait has been established.

Coal-packed biofilters show that the initial inoculum need not become the final process community. In Limbri et al. (2014), introduced *Methylosinus* did not remain dominant, whereas *Methylocystis*-related 16S rRNA gene sequences became abundant; this indicates selection but not active methane oxidation. Functional stability can coexist with taxonomic succession (Kim et al., 2014), supporting reproducible function rather than a fixed species list without proving functional redundancy.

Disturbance experiments provide a stricter test. Alphaproteobacterial methanotrophs differed in survival and recovery during methane feast–famine cycles, showing that maintenance and reactivation can rival maximum growth rate for intermittent sources (Cantera et al., 2023). Interaction networks also reorganized under stress (Ho et al., 2020), although network change alone is not a mechanism. A practical sequence is to enrich communities under realistic disturbances, identify recurrent active guilds, and reconstruct simplified consortia that retain experimentally supported functions.

## 5 Coupling microbial ecology with biofilter design

Biofilter design constrains which microbial traits persist. Compost and soil retain water and nutrients but can compact, mineralize, and develop nonuniform flow. Coal is operationally relevant but varies in pore structure, fines, wettability, nutrients, and indigenous microbiota. Inorganic carriers such as lava rock, fly-ash ceramsite, and autoclaved aerated concrete provide mechanical stability and tunable porosity but often require nutrient delivery. Composite beds may combine a water-retentive active phase with a rigid phase that preserves gas distribution.

Carrier effects should be resolved into adhesion, water retention, pore access, nutrient release, clogging, and gas transfer. Coal packing selected an attached methanotrophic community, while fly-ash ceramsite increased attached biomass and average elimination capacity relative to suspended cells (Limbri et al., 2014; Sun et al., 2020). Autoclaved aerated concrete sustained treatment near 1,000 ppmv, whereas porous cellulose beads supported treatment near 500 ppmv by a defined strain (Ganendra et al., 2015; He et al., 2025). These non-mine or laboratory observations make carrier properties transferable hypotheses rather than intrinsic performance rankings. Surface chemistry can affect adhesion, pore size can affect colonizable area and clogging, excess water can reduce gas-filled porosity, and mineral composition can alter pH and micronutrient availability under the conditions tested.

Methane transfer is often limiting because methane is sparingly soluble. In packed gas biofilters, gas-film, water-film, and intrabiofilm resistances may separate methane from active cells; their relative importance depends on carrier wetting, biofilm architecture, gas velocity, and local methane and oxygen demand. Moisture supports hydration but can lengthen diffusion paths or reduce air-filled pores. Thick biofilms can retain biomass and interactions while leaving inner regions substrate-limited. A candidate design target is therefore a hydrated, permeable biofilm with controlled thickness and spatial distribution that avoids excessive diffusion resistance, channeling, and pressure drop. Whether discontinuous colonization improves gas access without sacrificing active surface area requires direct testing.

Empty-bed residence time couples biological and physical constraints. Longer EBRT permits transfer and oxidation but increases reactor volume for high-flow VAM; higher gas velocity reduces footprint but can promote drying, channeling, or biomass detachment. Staged beds, parallel modules, structured packing, or intermittent irrigation are candidate configurations rather than established mine designs. Biotrickling recirculation can improve wetting, nutrient delivery, and heat control but adds gas–liquid transfer, pumping, and biomass-management demands. Designs should therefore be compared using methane oxidation evidence, pressure drop, energy use, and resource demand together.

Nutrient delivery requires similar restraint. Copper may favor pMMO expression in some organisms but becomes toxic in excess. Reduced nitrogen supports growth, whereas free NH_3_, hydroxylamine, or nitrite may inhibit oxidation depending on speciation, pH, and loading. Continuous rich medium can promote heterotroph overgrowth. Phosphorus, potassium, copper, and nutrient-solution timing have altered non-mine biofilter performance (Nikiema et al., 2010; Khabiri et al., 2020), but these observations do not establish mine-specific dosing rules. Pulsed or demand-responsive dosing is plausible; long-duration tests should determine how irrigation and biomass accumulation reshape community composition, pore structure, water distribution, and pressure drop.

## 6 Mine-scale deployment, operating thresholds, and safety

The mine-targeted evidence base remains small. Early gas-phase and ventilation-air biofilters established laboratory feasibility (Apel et al., 1991; Sly et al., 1993). Later studies examined coal-packed systems and mine-derived enrichments (Limbri et al., 2014; Jiang et al., 2016), while coal-contact, stress-acclimation, and reactor-device studies broadened the tested microbial consortia, carrier interactions, and concentration ranges (Zhou et al., 2023; Xie et al., 2025, 2026). No long-term field or pilot validation under authentic mine-gas operation was identified in the targeted search described above.

At larger scale, biological feasibility and operational/safety feasibility must be assessed separately. Site-specific limits should first be defined with mine operators from allowable ventilation disturbance and backpressure, gas composition and oxygen management, available footprint and residence time, water and nutrient logistics, condensate and biomass handling, hazardous-area equipment classification, and fail-safe bypass or diversion under process failure. Biological testing should then remain inside that operating envelope. A practicable EBRT is one that the available flow path and footprint can provide without unacceptable ventilation effects; acceptable pressure drop is the site allowance with margin for wetting, fouling, and biomass growth; and sustainable water or nutrient supply is demand that can be maintained across seasonal operation without unacceptable logistics or discharge. Oxygen addition or conditioning is no-go if it can create an unsafe gas composition or methane accumulation. If sustained oxidation requires operation outside any of these limits, the candidate should be redesigned or rejected. Numerical thresholds remain site- and jurisdiction-specific (United Nations Economic Commission for Europe (UNECE), 2025).

Landfill and compost systems provide transferable evidence rather than mine validation. Open-bed compost filters demonstrate long-term moisture management, thermal feedback, and community adaptation, including self-heating and thermophilic selection (Fjelsted et al., 2020; Gómez-Borraz et al., 2025; Avila-Nuñez et al., 2026). Their methane concentrations, gas composition, or EBRTs can differ substantially from mine streams. Mine-scale claims should therefore await authentic-gas pilots across seasons that jointly measure loading, elimination capacity, methane mass balance, pressure drop, water and nutrient use, disturbance recovery, safety performance, and net energy or climate benefit.

## 7 Discussion and future directions

Available evidence supports source-specific selection and validation of methanotrophic communities, but authentic-gas studies remain too scarce for universal design rules. Priority traits are activity at the target methane partial pressure, persistence through feed interruption, and function in a hydrated but permeable carrier. Nutrient economy and tolerance of temperature, moisture, dust, and co-contaminants should be evaluated alongside these traits. Reactor variables matter only insofar as they sustain these functions over time.

Four trade-offs organize the evidence: affinity versus volumetric productivity; defined cultures versus source-conditioned communities; moisture retention versus gas transfer and pressure drop; and active packed beds versus passive covers for different source classes. These context-dependent choices explain why optimizing an isolate, carrier, or single operating variable does not predict mine-compatible performance.

Common laboratory endpoints poorly predict mine-scale performance: batch maxima exclude gas-transfer constraints, high removal at long EBRT can imply impractical footprint, *pmoA* copy number does not establish current activity, and short stable runs miss ecological drift, nutrient depletion, drying, and clogging. A defensible minimum dataset includes inlet and outlet methane, flow, EBRT, loading, elimination capacity, temperature, water content, pressure drop, nutrient input, methane mass-balance checks, activity-resolved microbial measurements, and recovery from predefined disturbances. Techno-economic and life-cycle assessment is treated here as a downstream screening step rather than an established design criterion; current conclusions remain sensitive to measured performance distributions and site assumptions (Landgren et al., 2025).

Because mine-specific studies remain sparse and heterogeneous, this framework is a targeted narrative synthesis rather than a quantitative performance ranking. Differences in reactor metrics, concentration, EBRT, operating duration, pressure drop, and resource reporting preclude direct comparison of the values summarized in Table 1.

Testing the framework requires three linked tasks. First, source-realistic enrichment should expose mine-derived and environmental inocula to sub-percent methane, realistic temperatures, and repeated feast–famine cycles, followed by competition experiments that separate affinity from persistence. Second, activity-resolved analysis should pair time-resolved methane mass balances with at least one direct activity measure, such as RNA-based assays or stable-isotope probing; metagenomic or metatranscriptomic analyses can be added when they resolve specific population functions. Third, successful communities should be reduced to reproducible consortia and validated in modular reactors.

Consortium tests should pair methanotrophs with partners only when candidate functions such as vitamin provision, metabolite removal, or matrix modification have experimental support. Modular validation should target stable oxidation at acceptable pressure drop using structured packing, managed biofilm distribution, staged nutrients, and climate-appropriate operation. Open-source triplicate hardware tested at 0.5% CH_4_ illustrates how standardized modules can improve replication but does not constitute mine validation (Geiger et al., 2026). These remain testable priorities, not mine-scale design rules.

At the deployment boundary, a minimum reporting framework would make evidence cumulative. Reports should distinguish concentration from loading, state dry- or wet-gas basis, document acclimation and sampling, quantify replicate-bed uncertainty, and include methane mass-balance checks. Where feasible, field pilots should include untreated reference periods or validated non-biological controls because inlet variability can mimic apparent removal. Performance, moisture, pressure drop, activity, and community function should be interpreted together. Established evidence currently supports laboratory aerobic methane oxidation, source-dependent transfer constraints, and the need to report reactor performance beyond removal efficiency. Transferable evidence suggests useful roles for selected carriers, partner functions, moisture management, and disturbance tolerance, but these require mine-specific confirmation. Authentic-gas seasonal performance, durable functional microbiomes, site-specific operating thresholds, and mine-integrated safety performance remain unproven. Biofiltration should advance only when source-matched biological performance fits the independently defined operational and safety envelope; otherwise the appropriate outcome is redesign or no-go.
